# Assessment of a small-scale fishery: Lane Snapper (*Lutjanus synagris*) using a length metric method

**DOI:** 10.1371/journal.pone.0233479

**Published:** 2021-02-01

**Authors:** Liliana Sierra Castillo, Masami Fujiwara

**Affiliations:** 1 Department of Wildlife and Fisheries Sciences, Texas A&M University, College Station, Texas, United States of America; 2 Department of Ecology and Conservation Biology, Texas A&M University, College Station, Texas, United States of America; Hellenic Center for Marine Research, GREECE

## Abstract

Small-scale fisheries are hard to assess because of the limited availability of data. Therefore, a method requiring easy-to-obtain catch-data is important for the assessment and management of small-scale fisheries. The objective of this study was to assess the effect of fishing gear selectivity on a length-based metric method proposed by Froese by estimating three indicators using catch-data from Lane Snapper (*Lutjanus synagris*) collected in Honduras. These indicators are (1) the percentage of mature individuals in the catch, (2) the percentage of fish within the range of estimated optimal lengths to be captured, and (3) the percentage of fish larger than the optimal length. These indicators determine the level of overfishing. The indicators were estimated separately for catch-data corresponding to gill nets, and each indicator was estimated with and without selectivity correction. Selectivity and mesh sizes of the fishing gear had a major impact on the estimation of indicators 1 and 2. As for indicator 3, it consistently showed a high level of exploitation. The three estimated indicators suggested that the Lane Snapper fishery in Honduras is experiencing overfishing. Overall, the method appears to be promising for the assessment of small-scale fisheries, but it should be used cautiously.

## 1. Introduction

The status of small-scale fisheries around the world is uncertain because of a lack of adequate data [1–4] (Ault et al., 2008; Babcock et al., 2013; Babcock et al., 2018; Worm et al., 2009). These fisheries are often not assessed or are assessed inadequately [5–9] (Aschenbrenner et al., 2017; Carruthers et al., 2014; Froese, 2000; Froese et al., 2012; Levin et al., 2006). Because the stock-assessment models used for the assessment of fisheries are designed for large-scale fisheries, requiring a large amount of high-quality data [10] (Cope & Punt, 2009), it is often very difficult to assess small-scale fisheries using these models [2, 3, 8] (Babcock et al., 2013; Babcock et al., 2018; Froese et al., 2012). Nevertheless, small-scale fisheries are very important globally and still need to be assessed [11–13] (Andrew et al., 2007; Hilborn et al., 2003; Pauly, 1997).

In order to assess the status of small-scale fisheries, models used to assess large-scale fisheries (such as yield per recruit) are often applied; however, because of the complexity inherent in the models and the data needed to use them, the results are often not satisfactory [14] (Froese, 2004). Thus, it has been suggested that the assessment methods for small-scale fisheries should depend on a simple method using the data that are easy to obtain, such as size/age frequency data [4, 15–17] (Freitas et al., 2014; Orensanz et al., 2005; Punt et al., 2001; Worm et al., 2009). The analysis of length frequency data can provide important information about shifts in the population age/size structure, which can be indicative of overexploitation [3] (Babcock et al., 2018).

One method that uses length frequency data to assess the level of exploitation for a stock is the estimation of exploitation rates. Exploitation rates can help inform fishery managers of the level of fishing pressure occurring in the fishery, and thus help them in managing the fishery accordingly. The length metric method proposed by [14] Froese (2004) is one method that is simple and depends on commonly available length frequency data. The method utilizes length data from catches to estimate three indicators of overfishing: (1) the percentage of mature fish present in the length frequency data, (2) the percentage of fish caught within an optimal length, and (3) the percentage of “mega spawners” present in the length frequency data. “Mega spawners” can be defined as fish that are larger and older (when compared to the fish in the catch), and they are important to the fishery because it is assumed that the larger the fish it tends to be more fecund and thus has a greater ability of replenishing the fishery [14, 18] (Froese, 2004; Gwinn et al., 2015). These indicators are designed to examine the size distribution of fish in the ocean. The logic behind them is that (1) there should be a high percentage of mature individuals (individuals that are above the length where at least 50% of the population has reproduced at least once) in the ocean to avoid recruitment overfishing; (2) there should be a high percentage of individuals that are considered to be within the range of optimal lengths, which are the lengths at which the highest yield can occur; and (3) the percentage of “mega spawners” present in the ocean should be between 20% and 40% in order to conserve large and mature individuals, and thereby avoid growth overfishing [5, 10, 14] (Aschenbrenner et al., 2017; Cope & Punt, 2009; Froese, 2004). Its simplicity makes the method very attractive to fishery managers [5, 9, 10, 19, 20, 21] (Aschenbrenner et al., 2017; Busilacchi et al., 2012; Cope & Punt, 2009; Cury & Christensen, 2005; Francis et al., 2007; Lewin et al., 2006).

These indicators provide important information for fishery assessment [3] (Babcock et al., 2018). Overfishing tends to occur when the catches in a given period exceed the catches that surpass a desired threshold, such as maximum sustainable yield or maximum yield-per-recruit [22] (Hilborn & Hilborn, 2011). Overfishing may be categorized into recruitment overfishing, which occurs when recruitment is reduced, or growth overfishing, which happens when too many small fish are caught. Indicators 1 and 3 determine these two types of overfishing that might be occurring in the fishery [23] (Sissenwine & Shepherd, 1986). Indicator 2 helps to achieve the maximum sustainable yield of the fishery [14] (Froese, 2004).

Even though using these indicators might be promising for the assessment of small-scale fisheries, one major drawback is the underlying assumption that the catch length composition is representative of the fish in the ocean [2, 10] (Babcock et al., 2013; Cope & Punt, 2009). The data in small-scale fisheries are usually fishery dependent. This is particularly problematic when the catches do not represent the natural populations because of gear selectivity. Gill nets are one of the most size-selective fishing gear, and the resulting catches may not represent the actual structure of a population [24, 25] (Doll et al., 2014; Gulland, 1987), and gill nets are commonly used in small-scale fisheries [26–31] (Acosta, 1994; Hamley, 1975; Lobyrev & Hoffman, 2018; McClanahan & Mangi, 2004; Reis & Pawson, 1992; Shoup & Ryswyk, 2016).

Developing an approach for assessing and managing small-scale fisheries with simple methods using readily available data is critically important. The objective of this study was to determine if the assumption behind the method proposed by [14] Froese (2004) is appropriate for the Lane Snapper (*Lutjanus synagris*) from a small-scale fishery in Honduras. We also determine the status of the population of the Lane Snapper (*Lutjanus synagris*) under current management practices. We will use length data collected in Tela, Honduras, as a case study and discuss the adequacy of the length-based method for the assessment of small-scale fisheries.

## 2. Methods

### 2.1 Collection of data

**Fig 1:**
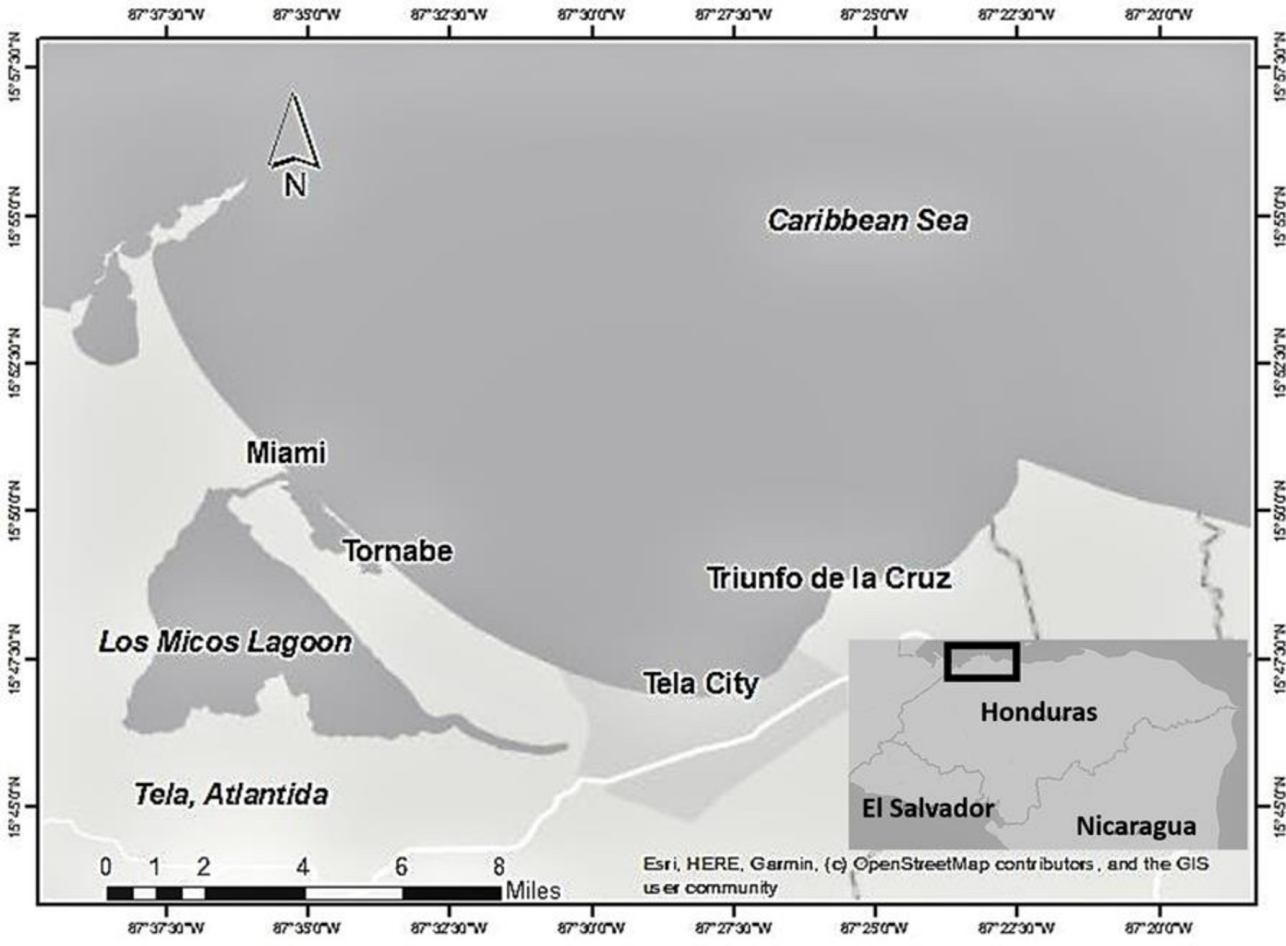
Map of Tela on the northern coast of Honduras and the villages surveyed: Miami, Tornabe, Tela Town, and Triunfo de La Cruz.

Sampling was conducted in Tela, Honduras (Fig 1), by the Coral Reef Alliance, the World Wildlife Fund (WWF), and the National University of Honduras (UNAH) as part of a project from the Coral Reef Alliance in coordination with local Honduran authorities. For this project, no ethical permissions were required because the surveys were done in coordination with local Honduran authorities who manage the areas in Tela. Tela is a coastal town located on the northern coast of Honduras. Data was collected by surveying fishers in the villages of Tornabe, Triunfo de la Cruz, Tela Town, and Miami from 2015 to 2017.

These towns were selected over others because of accessibility and a greater number of fishers (1–4 fishers per fishing boat and~ 5–10 fishing boats per town). For each day, the fishers were surveyed in the order of arrival to the coast or port in each village. When multiple fishers arrived closely together in time, some fishers departed before being surveyed. In these cases, these fishers were not included in the surveys. Sometimes the weather did not permit for fishing; as a result, the amount of surveys was not consistent among days and towns. However, these inconsistencies should not cause any bias in size distribution in our sample.

In total 194 surveys were carried out amongst the different villages and they were done ~2 days per week in each village (alternating villages). In each survey, species, fork length (centimeters), and weight (grams) of each fish caught were recorded. The duration of the fishing trip (minutes) and characteristics of the fishing gear being used (type of gear, size of gear, and mesh size for gill nets) were also collected. Then, the catch (the number of fish) per unit effort (minutes) for each gear category (gill net mesh size 2″ or 3″) was estimated. These estimations assume catchability remained constant, and that the fishing area did not change over time. Even though the fishery is a multispecies fishery (each catch consisted of different species), Lane Snapper (*Lujtanus synagris*) caught with gill nets of 2″ and 3″ mesh sizes were solely taken into consideration for this study because of the importance of this species for local economies in the area and because they were the most common fishing gear types targeting the species in the fishery [32] (Carbajal et al., 2017). In the different catches reported by the fishermen, most of the time there was always at least one Lane Snapper reported (this was particularly true in the villages of Tornabe, Tela and Miami). We assume that there is no significant discard of small individuals occurring and that these catches are a representation of our stock for the area. We further assume that the population is under stable size distribution although the population may be growing or declining its abundance over time. We note that other fishing gears, as well as gill nets of other mesh sizes, were also used in the fishery. Therefore, the fishing mortality is not coming solely from gill nets of 2″ and 3″ mesh.

### 2.2 Biology and ecology of the Lane Snapper (*Lutjanus synagris*)

The Lane Snapper, *Lutjanus synagris* lives in coastal reefs and adjacent habitats throughout the tropical Atlantic. This species has been reported to reach sizes up to 50 cm. The juveniles of this species are usually found in nearshore habitats such as estuaries and rocky reefs. The adults tend to live in deeper reefs, and it is reported that when they reach sexual maturity (~ 20 cm of length) they form aggregations to spawn [5, 15] (Aschenbrenner et al., 2017; Freitas et al., 2014). For our study, the oldest individuals reached an age of 8 years; however, studies in other areas report individuals can reach an age of 10 years [15] (Freitas et al., 2014).

### 2.3 Analysis of data

For this study, the length-based method proposed by [14] Froese (2004) was applied to assess the Lane Snapper (*Lutjanus synagris*) fishery in Tela, Honduras. The method consists of the estimations of three indicators that provide the information needed for the assessment of the fishery. The main assumption behind this method is that the length composition of the catch is representative of the length composition of the fish in the ocean. Each indicator was determined for the data belonging to Lane Snapper (*Lutjanus synagris*) caught with gill nets of 2” and 3” mesh size. The selectivity bias associated for each mesh size was corrected by fitting different distribution curves (a.k.a selectivity curves) to the length-specific CPUE of each mesh size, this was done using a maximum likelihood method (as described in more detail in [33] Sierra Castillo et al., (2020)).

### 2.4 Percentage of mature fish present in the length frequency data

The catches of healthy fisheries are expected to include a high percentage of mature individuals [5, 14] (Aschenbrenner et al., 2017; Froese, 2004). For this indicator, we determined the amount and percentage of individuals that were above the *L*_50_, which was the length where 50% of the individuals were able to reproduce. For this study, the average of the different values for *L*_50_ reported in the study in Roatan, Honduras, was used [34] (Berthou et al., 2001).

#### a. Percentage of fish caught within an optimal length

The optimal length is the length of individuals caught where the maximum yield is achieved [14] (Froese, 2004), and it was estimated using the expression given by [35] Beverton (1992):

$$L_{opt}=\frac{3L_{\infty }}{\left(3+\frac{M}{k}\right)}$$

where *L*_∞_ is the maximum length that fish in the population would reach if they were to grow indefinitely, *k* is the growth parameter, and *M* is the natural mortality. For this study, we assumed that the values of *L*_∞_ of 51.6 cm and *k* of 0.23 estimated for Puerto Rico for Lane Snapper (*Lutjanus synagris*) were the same for our population and that they remained constant through the study [36] (Acosta & Appeldoorn, 1992). Different natural mortalities estimated for Lane Snapper (*Lutjanus synagris*) by [33] Sierra Castillo et al., (2020) were used to assess the impact on the optimal length estimation (Appendix A). A range ±10% of the optimal length—as proposed by [2, 10, 14] Babcock et al., (2013), Cope and Punt (2009), and Froese (2004)—was used to determine the percentage of the catch that falls within the optimal length.

#### b. Percentage of fish caught that are “mega spawners”

We estimated the percentage of fish that were larger than the optimal length plus 10%, and considered it to be the percentage of “mega spawners” present in the catch [14] (Froese, 2004). Because we have different *L*_*opt*_ ranges (according to the natural mortality *estimates* used), we used the highest *L*_*opt*_ value and estimated the percentage of “*mega spawners*”. We did not include the *L*_*opt*_ estimated based on the method of Peterson *and Wroblew*ski (*L*_*opt*_ > 35 cm) because of its poor performance [33](Sierra Castillo et al., 2020). The expected percentages of “mega spawners” should be between 30% and 40% for a healthy stock without overfishing. A percentage of “mega spawners” lower than 20% indicates potential overfishing [3, 14] (Babcock et al., 2018; Froese, 2004).

## 3. Results

### 3.1 Indicator 1: Percentage of mature fish in the length distribution of the catches

**Fig 2:**
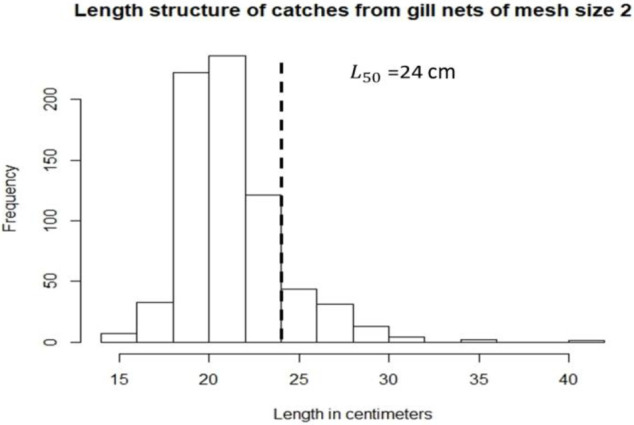
Length distribution of Lane Snapper (*Lutjanus synagris*) caught with gill nets of mesh size 2” (n = 714); dashed line demonstrates the *L*_50_ used and the frequency of fish that are above and below this.

**Fig 3:**
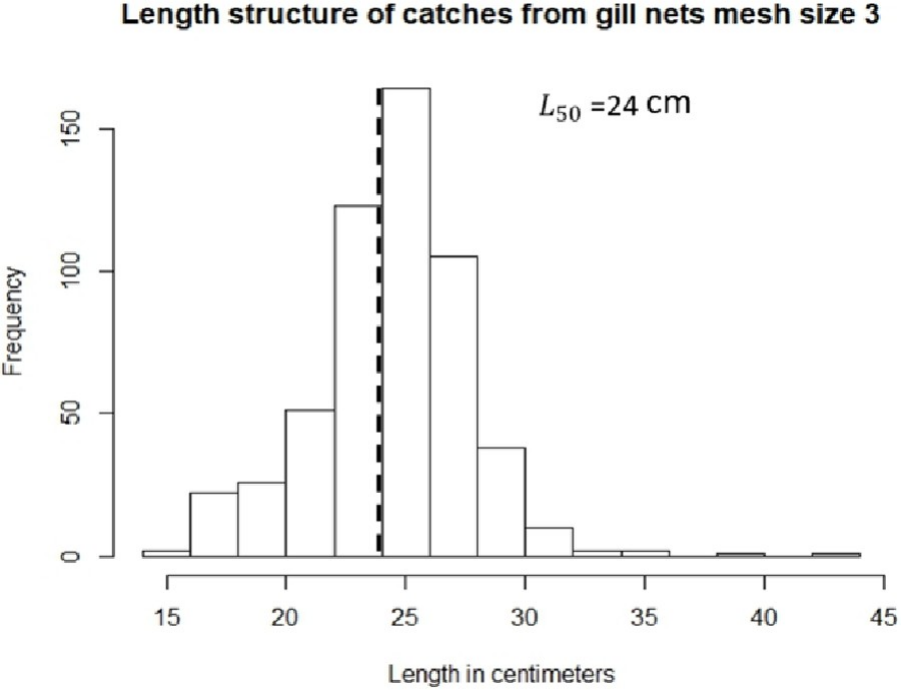
Length distribution of Lane Snapper (*Lutjanus synagris*) caught with gill nets of mesh size 3” (n = 548); dashed line demonstrates the *L*_50_ used and the frequency of fish that are above and below this.

Figs 2 and 3 demonstrate the length frequency distribution of the different catches with 2” and 3” mesh size gill nets without selectivity correction. For mesh size 2” (Fig 2), the percentage of mature individuals that are being caught is 13% (when using an *L*_50_ of 24 centimeters, n = 714), which results in 87% of immature fish in the catch with this mesh size. On the other hand, for mesh size 3” (Fig 3), the percentage of mature individuals reported in the catches is 59% (41% of immature individuals, n = 548). For this indicator, the percentage of mature individuals should be high [14] (Froese, 2004). As seen in Figs 2 and 3, gill nets of mesh size 3” catch the percentage of mature individuals close to what is recommended, whereas gill nets of mesh size 2” catch a large amount of immature fish.

After correcting for selectivity (i.e., for 2” and 3” mesh sizes) and obtaining the percentage of individuals that are above *L*_50_ (24 cm), there are around 21% of mature individuals in the stock in the ocean.

### 3.2 Indicator 2: Percentage of fish caught at an optimum length

**Fig 4:**
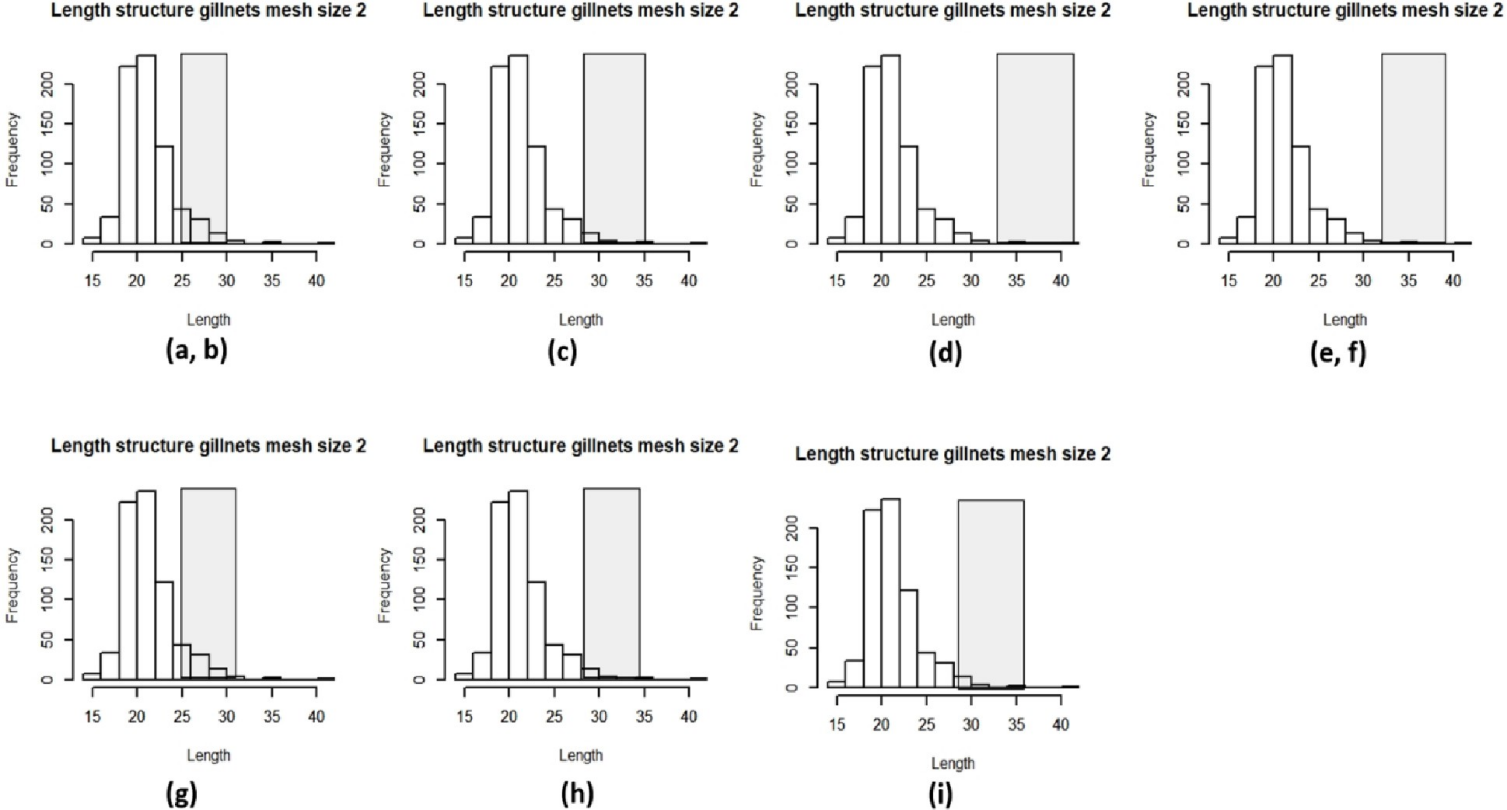
Length frequency distributions of fish caught with mesh size 2” and the optimum range of lengths, each letter represents the different values of natural morality used as seen in Table 1.

**Fig 5:**
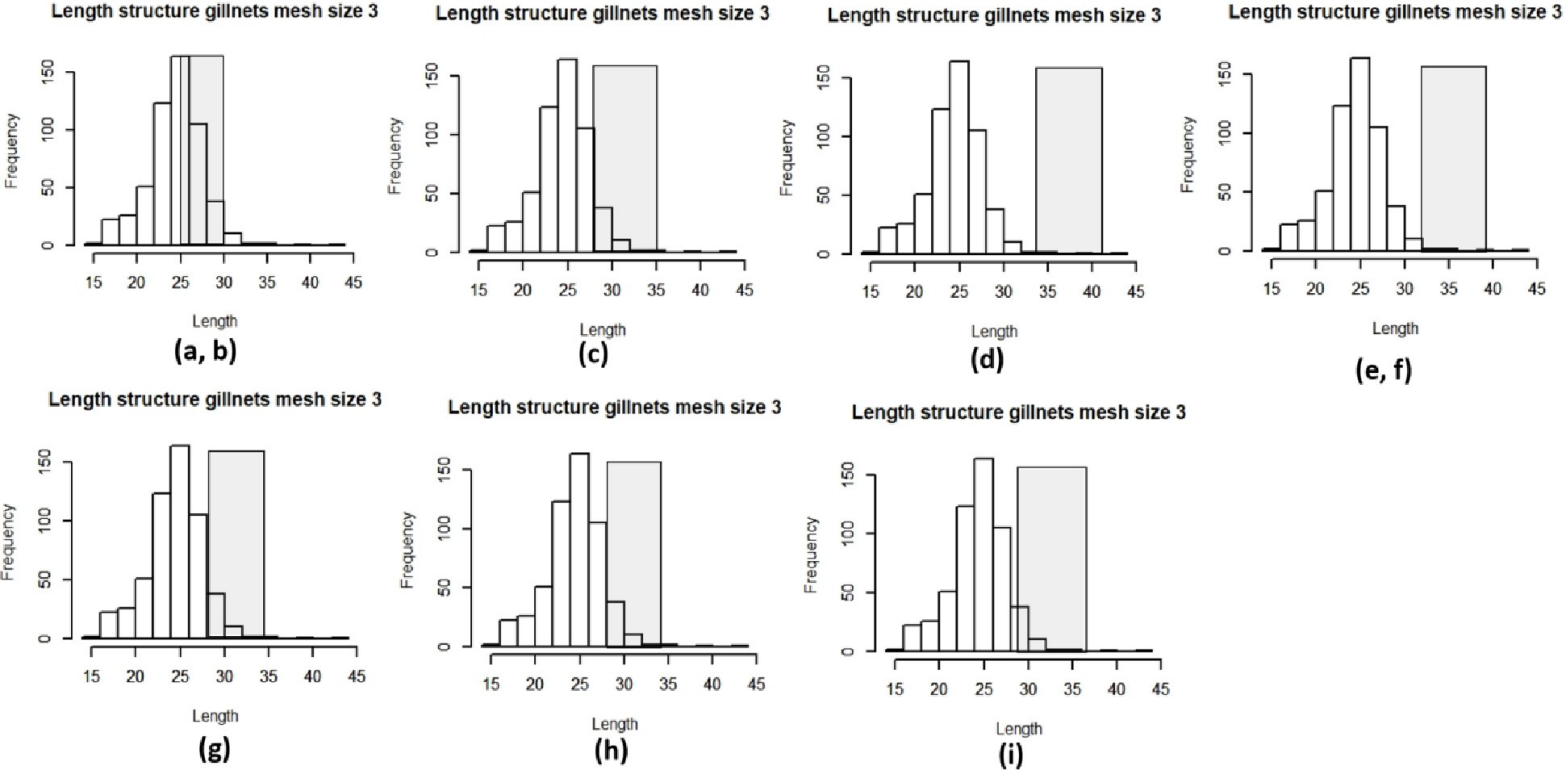
Length frequency distributions of fish caught with mesh size 3” and the optimum range of lengths, each letter represents the different values of natural morality used as seen in Table 1.

Figs 4 and 5 show the length frequency for each mesh size and the range of lengths considered to be optimum for capture. The different natural mortalities (letters a-i represent the natural mortality estimates used in Figs 4 and 5) used had an impact on these ranges and thus on the percentages of individuals that were captured at an optimum length (Table 1). As with indicator 1, gill nets of mesh size 2” have low percentages of capturing fish that are in optimum length as seen in Table 1. On the other hand, gill nets of mesh size 3” have higher percentages of individuals that are being captured in the optimum lengths.

**Table 1:** Estimated range of optimum lengths and the percentage of individuals caught at optimum length with different mesh sizes under different values of assumed natural mortality, with and without selectivity correction.

Method used for the estimation of natural mortality as seen in Sierra Castillo et al., (2020) & letter used to represent them	Natural mortality estimates	Range of optimum lengths	Percentage of individuals caught at optimum length without selectivity correction (mesh size 2”)	Percentage of individuals caught at optimum length with selectivity correction (mesh size 2”)	Percentage of individuals caught at optimum length without selectivity correction (mesh size 3”)	Percentage of individuals caught at optimum length with selectivity correction (mesh size 3”)
Lorenzen using average of weights **(a)**	0.60	24 cm- 30 cm	12.32%	19.93%	56.12%	20.46%
Lorenzen using median of weights **(b)**	0.61	25 cm- 30 cm	12.32%	19.93%	56.12%	20.46%
Lorenzen using midpoint of 8 years **(c)**	0.44	28 cm- 35 cm	4.48%	11.42%	17.73%	5.25%
Peterson & Wroblewski using average of weights **(d)**	0.24	34 cm- 42 cm	0.42%	4.66%	0.55%	0.22%
Peterson & Wroblewski using median of weights **(e)**	0.32	32 cm- 39 cm	0.56%	6.06%	1.46%	0.50%
Peterson & Wroblewski using midpoint of 8 years **(f)**	0.32	32 cm- 39 cm	0.56%	6.06%	1.46%	0.50%
Sekharan 8 years **(g)**	0.56	25 cm- 31 cm	12.46%	20.98%	57.40%	20.85%
Sekharan 10 years **(h)**	0.46	28 cm– 34 cm	4.48%	11.42%	17.55%	5.20%
FishLife **(i)**	0.41	29 cm -36 cm	2.66%	12.00%	9.51%	2.77%

When correcting for the selectivity that gill nets have, the results indicate that approximately 20% of fish in our stock in the area would be at the optimum length to be caught (Table 1).

### 3.3 Indicator 3: Percentage of fish caught that are considered to be “mega spawners”

The percentages of mega spawners that are being caught with both mesh sizes (2” and 3”) were very low (0.14% and 0.37%, respectively) without correcting for selectivity. Similarly, when taking the selectivity into consideration, there are about 0.3% of mega spawners in the population in the area. For this indicator, the percentage of mega spawners in the catch should be greater than 20% for a stock to be considered healthy [14] (Froese, 2004).

## 4. Discussion

The method proposed by [14] Froese (2004) to assess a fish stock may not be adequate for the assessment of small-scale fisheries without correction for the size-selectivity of the fishing gear. The method assesses the size distribution of the stock in the ocean based on size distribution in the catch. Therefore, the assumption behind this method is that there is no gear selectivity affecting the catches of the fishermen, and thus the catches are representative of the fish stock [3, 10] (Babcock et al., 2018; Cope & Punt, 2009). However, most small-scale fisheries use size-selective fishing gear, and this was the case with our data from the small-scale fishery in Honduras.

When we used the catches from fishing gear that targets smaller fish (e.g., Lane Snapper data collected with 2” mesh size gill nets from Honduras) to estimate the indicators without consideration of the impact of the selectivity, we will obtain lower values for indicators 1 and 2 (Table 2). Using these indicators without selectivity correction to make management decisions can be problematic [10] (Cope & Punt, 2009). For example, indicator 1 without selectivity correction in our study suggests that only 13% of the stock is mature (using the catch as a representation of the stock); however, the same indicator after correcting for gear selectivity suggests that 33% of the stock is mature.

**Table 2:** Estimation of indicators with and without selectivity correction using different methods for the estimation of natural mortality (represented with letters), catches from mesh size 2” gill nets and 3” gill nets.

Size of mesh size gill nets	Indicator and when needed method used for the estimation of natural mortality as seen in Sierra Castillo et al., (2020) represented by a letter as seen in Table 1	Estimation of indicators not correcting for selectivity	Estimation of indicators correcting for selectivity
2”	Indicator 1	13%	33%
2”	Indicator 2 **(a)**	12.32%	19.93%
2”	Indicator 2 **(b)**	12.32%	19.93%
2”	Indicator 2 **(c)**	4.48%	11.42%
2”	Indicator 2 **(d)**	0.42%	4.66%
2”	Indicator 2 **(e)**	0.56%	6.06%
2”	Indicator 2 **(f)**	0.56%	6.06%
2”	Indicator 2 **(g)**	12.46%	20.98%
2”	Indicator 2 **(h)**	4.48%	11.42%
2”	Indicator 2 **(i)**	2.66%	12%
2”	Indicator 3	0.14%	0.3%
3”	Indicator 1	59%	21%
3”	Indicator 2 **(a)**	56.12%	20.46%
3”	Indicator 2 **(b)**	56.12%	20.46%
3”	Indicator 2 **(c)**	17.73%	5.25%
3”	Indicator 2 **(d)**	0.55%	0.22%
3”	Indicator 2 **(e)**	1.46%	0.50%
3”	Indicator 2 **(f)**	1.46%	0.50%
3”	Indicator 2 **(g)**	57.40%	20.85%
3”	Indicator 2 **(h)**	17.55%	5.20%
3”	Indicator 2 **(i)**	9.51%	2.77%
3”	Indicator 3	0.37%	0.3%

On the contrary, with fishing gear that targets larger sizes of fish (e.g., Lane Snapper data collected with 3” mesh size gill nets from Honduras), the catch—and thus length—structures consist of larger age classes. As a result, when selectivity is not taken into consideration, the estimation of indicators 1 and 2 will be higher (Table 2). Indicator 1 in our study (without selectivity correction) suggests that 59% of the individuals in our catch are mature; this may suggest that the stock is healthy. However, the percentage of mature individuals, when taking selectivity into account, is 21%, and thus recommending the use of mesh size 3” (or larger) might affect the older age classes of the stock. This will cause problems when interpreting the indicators to make management recommendations [2, 3, 10] (Babcock et al., 2013; Babcock et al., 2018; Cope & Punt, 2009).

Although indicator 3 values for both mesh sizes (2” and 3”) are lower when compared with the indicator estimated after correction for gear selectivity (Table 2), the estimation can also be higher if gear with a much larger mesh size is used. It can potentially suggest that the stock may be healthy, but it does not reflect the true condition of the stock [3] (Babcock et al., 2018).

The status of the Lane Snapper in Tela, Honduras, based on the three indicators after correcting for the size-selectivity of the gill nets, is an overfished stock. With indicator 1, the percentage is 21%, which suggests that a small percentage of fish are mature. Additionally, indicator 3 shows that the percentage of mega spawners is currently 0.3% when it should be greater than 30%. Therefore, recruitment overfishing and growth overfishing are occurring with the stock [8, 14, 37] (Froese, 2004; Froese et al., 2018; Froese et al., 2012). Agreeing with [33] Sierra Castillo et al., (2020), there seems to be high fishing pressure (resulting in high fishing mortalities), which is currently causing the overfishing. Because of this, we recommend that managers work alongside fishermen to enforce the existing fishing regulations, such as the ban on gill nets of mesh size 2”. Even though they are illegal to use in Honduras (as stated by law), there are many fishermen still using them. It may be difficult to enforce the ban of mesh size 2” gill nets in Honduras because of the unique sociological and economic characteristics surrounding the fishery, but we believe it is possible—by working side by side with the fishers and local organizations—to implement this recommendation [32] (Carbajal et al., 2017)

At the same time, the fishing pressure on the older age classes should be reduced. To achieve this goal, we recommend closing the areas where older age classes of Lane Snapper spend most of their time and protecting these areas from gill net fisheries. This can be implemented as part of the regulations of the marine protected area (MPA) recently established in the area. In addition, the overall fishing effort should be decreased by reducing the number of gill nets the fishers can use in each fishing trip.

In general, the method proposed by [14] Froese (2004) might be very appealing to fishery managers due to its simplicity and accesibility; however, the results should be interpreted with caution. Without consideration of the selectivity of fishing gear, the indicators may be overestimated or underestimated. However, we also understand that the simplicity of the method, which is the major advantage of it, is undermined by including an additional step in calculating the indicators considering selectivity. Furthermore, there may not be adequate data to correct for gear selectivity; however, our study suggests it is very important to correct for the selectivity bias. When it is necessary to use the indicators estimated without correcting for gear selectivity, it is important to know that indicators will have higher values when the fishing gear selects smaller fish and lower values when the gear selects larger fish. Thus, the management decision should be adjusted accordingly.

The biases caused by gear selectivity are in addition to potential biases and other uncertainty associated with parameters taken from other studies. For example, uncertainty exists in the parameters associated with the von Bertalanffy growth curve used in our study. The proposed method is also limited because although it can be used to determine the current fishing level (i.e. overfishing or not), it is not designed to determine the past fishery effect (i.e. overfished or not). Consequently, it may be necessary to take a precautionary approach when this particular method is used especially if the fishing gear is highly selective to certain sizes. Overall, the length metric method proposed by [14] Froese (2004) might seem promising for small-scale fisheries because the data that is needed can be easily obtained, but we recommend the method be used cautiously.

## Supporting information

S1 Data (XLSX)
